# Effects of the White Sulphur Water of Greenbrier, Virginia, in Inebriation

**Published:** 1872-03

**Authors:** J. J. Moorman

**Affiliations:** Professor of Hygiene and Medical Jurisprudence in Washington University, Baltimore, Maryland, and Physician to the Greenbrier White Sulphur Springs, Virginia


### EFFECTS OF THE WHITE SULPHUR WATER OF GREENBRIER, VIRGINIA, IN INEBRIATION.

BY J. J. MOORMAN, M.D.,
Professor of Hygiene and Medical Jurisprudence in Washington University, Baltimore, Maryland, and Physician to the Greenbrier White Sulphur Springs, Virginia.

A popular political journal has recently called public attention to a case of long-continued *inebriation* of a gentleman, which has been, as is believed, effectually *cured* by a persevering use of the *White Sulphur water.* This gentleman, as we are told, had been a hard and excessive drinker for *five years*, and had suffered *three severe attacks of mania potu.* This case, it is said, has elicited the attention of eminent medical men in New York, and writers in reference to it are suggesting new hopes to the inebriate from the use of the same means.

Without comment upon the case referred to, I desire to state what my long administration of these waters authorizes me to regard as their reliable influences in *habitual inebriation.* That a proper and continuous use of them exerts some power in this direction, I have no doubt.

I by no means claim that the White Sulphur waters should be regarded as a *specific* against either the love or intemperate use of alcoholic drinks, but simply that a proper use of them is a decided preventive of that feeling of *necessity* or *desire* for the use of strong drink which drives the inebriate to use it in despite of his judgment to the contrary; in other words, that their proper use *allays* or destroys the appetite or *nervous craving* for ardent spirits, and to such extent that even the habitual drinker and confirmed inebriate feels little or no desire for them *while he is properly using the waters.*

During my long residence as Physician to these Springs, I have witnessed hundreds of cases fully justifyining the above statement. This peculiar influence of the waters depends—*first*, upon the action of the *sulphuretted hydrogen gas* that abounds in them, and which is an *active nervine stimulant*, and as such supplies the want the inebriate feels for his accustomed alcoholic stimulant; and *secondly*, it depends upon the *alterative* influence exerted by the waters upon the entire organism. While by its alterative power the entire animal structure is brought into natural and harmonious action, there is a consequent subsidence of the *cerebral* and *nervous irritation* which always prevails in the drunkard—the abatement of which enables him to exert a moral power greater than he could before, and sufficient to overcome the lessened demand which his old habit, if he retain it in any degree, now makes upon him.

In the initiatory or forming state of intemperance, the free use of thls water may be much relied upon to modify, or entirely prevent, the *temptation* for strong drink; and even in the confirmed stage, its persevering use may inaugurate a state of the system that may materially aid the sufferer in overcoming the hurtful habit of intemperance. Indeed, if the habitual drinker can be prevailed upon to use the water properly for some ten days, to the entire exclusion of all alcoholic stimulants, he will have, for the time at least, but little alcoholic temptation to resist.

Of course I will not be so misunderstood as that it shall be supposed that I designed even to intimate an opinion that this water is a *sure* and *permanent cure* for either *absolute* or *threatened inebriation.* All that I design to assert in this connection is, *that a proper and continuous use of the water will very essentially aid the intemperate drinker* to lay aside the inebriating cup, and return to soberness. The *will* of the excessive drinker must necessarily concur, to some extent, with any effort successfully made for his relief. But while this is so, an auxiliary agent, as innocent in itself as sulphur-water, that can so far satisfy the *nervous cravings* of the votary of strong drink as to give him increased power to resist his morbid habit, while at the same time his general health is improved, well deserves, I conceive, the attention of all who need assistance in this direction.

It would be irrational for the inebriate to expect to be cured of his hurtful habit by simply visiting the Springs and drinking its waters, however freely, *and at the same time* (which has been the custom of some) to drink freely, also, of alcoholic liquors. Such a course could be of no service whatever. Stimulants of whatever kind, in such cases, must be abstained from while the water is establishing its peculiar action upon the system. This effected—which can ordinarily be accomplished in ten or twelve days—the success of future perseverance in the use of the water is *hopeful*, and easily, thereafter, under the control of the individual who is seeking relief.

---

Carbolic Acid Paper.—C. Homburg, of Berlin, has introduced, for disinfecting purposes, a paste-board saturated with crude carbolic acid, so that each square foot contains one hundred grammes. The atmosphere may be impregnated with the acid by suspending a suitable sheet in the rooms, the large surface of the paper favoring evaporation. For the disinfection of spittoons, urinals, ped-pans, and the like, small pieces of the paper are sufficient. The article is sold retail in sheets measuring about seven square feet, at twenty-five cents.—*Pharm. Centr. Halle*, 1871.

